# Graves' Disease Presenting as Painful Goiter: A Case Report and Review of the Literature

**DOI:** 10.7759/cureus.2765

**Published:** 2018-06-08

**Authors:** Amir Shahbaz, Kashif Aziz, Muhammad Umair, Mohaddeseh Sharifzadeh, Issac Sachmechi

**Affiliations:** 1 Internal Medicine, Icahn School of Medicine at Mount Sinai Queen Hospital Center, New York, USA

**Keywords:** graves' disease, thyroiditis, thyroid

## Abstract

A painful and tender thyroid gland is a rare phenomenon in Graves' disease. We present a case of 31-years-old Guyanese female who was admitted to the hospital with complaints of neck pain for the past few days. She also reported increase appetite for a few weeks; she did not have palpitations, heat intolerance, or muscle weakness. The presence of hyperthyroidism with elevated TSI suggested that the patient had Graves' disease. Erythrocyte sedimentation rate (ESR) and C-reactive protein (CRP) were within normal limit, thus the diagnosis of thyroiditis was effectively ruled out. This is a rare case of a painful and tender thyroid gland in Graves' disease without the evidence of sub-acute thyroiditis.

## Introduction

Graves' disease is the most common cause of hyperthyroidism. It is characterized by a diffusely enlarged thyroid gland with a high titer of thyroid-stimulating antibodies (TSI) and an increased uptake of radioiodine by the thyroid gland. A painful and tender thyroid gland is an uncommon feature of Graves' disease [1-3]. We present a case of Graves' disease where the patient primarily presented with complaints of pain and tenderness in the region of the thyroid gland without evidence of sub-acute thyroiditis.

## Case presentation

A 31-year-old Guyanese female presented to the emergency department (ED) complaining of odynophagia and neck pain for few days. She also reported an increased appetite over the past few weeks. She was otherwise healthy in the weeks leading up to her visit to the ED. She denied any history of palpitation, heat intolerance or muscle weakness. On physical examination, her blood pressure was 136/72 and pulse was 112/min with a sinus rhythm. Her skin was warm and dry. There was no exophthalmos or lid lag. The thyroid gland was diffusely enlarged and tender on palpation. A bruit was heard on bilateral lobes of the thyroid gland. She had fine tremors on outstretching of hands. Thyroid-stimulating hormone (TSH) was suppressed 0.03 mI U/ml (0.34- 5.6), while free thyroxine (free T4) 5.37 ng/dl (0.58-1.64), free triiodothyronine (free T3) 767.5 ng/dl (6.09-12.2), and total thyroxine (T4) 27.88 mcg/dl (6.09-12.2) were elevated. The suppressed TSH and elevated thyroid hormones were consistent with hyperthyroidism.

Further workup also demonstrated elevated levels of thyroid stimulating antibody (TSI) 221 (<125). Thyroid peroxidase antibody (TPO Ab) >1000 IU/mL (<35) and thyroglobulin antibody (TgAb) 279.0 IU/ml (<20) were also positive. Erythrocyte sedimentation rate (ESR)and C-reactive protein (CRP) were 10mm/hr (10-20) and <0.01 mg/dl (0-0.8), respectively. The radioiodine thyroid uptake scan (RAIU) was not done, as the patient was given iodinated contrast in ED for computed tomography (CT) of the neck. The presence of an enlarged thyroid gland and elevated thyroid hormones with positive TSI suggested that the patient had Graves' disease. As ESR and CRP were within normal limit, the diagnosis of subacute thyroiditis was effectively ruled out.

The patient was discharged from the hospital on methimazole 30 mg daily and propranolol 20 mcg three times a day. During the third month follow up visit, she had no pain in the thyroid and was clinically euthyroid on antithyroid medication. This was a rare case of Graves' disease presenting as a painful and tender thyroid gland without the evidence of subacute thyroiditis. The cause of pain, in this case, remains uncertain.

## Discussion

Graves' disease is the most common cause of hyperthyroidism and usually presents as a painless goiter. It is an autoimmune disease which results from the production of IgG autoantibodies against TSH receptors of thyroid follicular cells. These antibodies lead to follicular cell hyperplasia, resulting in diffuse enlargement of the thyroid gland and an increased production and secretion of thyroid hormones. Unrecognized Graves' disease has a negative impact on the quality of life and makes the patient vulnerable to an increased risk of psychosis, tachyarrhythmia, and cardiac failure [4]. Thyroiditis is the inflammation of thyroid gland due to numerous etiologies. The most common cause of sub-acute thyroiditis is various viral infections. Tenderness of gland and pain are a common feature of sub-acute thyroiditis. It is characterized by elevated levels of inflammatory markers i.e., ESR and CRP [5]. The initial presentation with thyroid pain and tenderness in our case suggested sub-acute thyroiditis; the normal ESR and CRP refuted that diagnosis. Hashimoto’s thyroiditis may rarely follow an episode of sub-acute thyroiditis [6]. The clinical picture of our patient did not support the diagnosis of Hashimoto's thyroiditis, which is a chronic painless thyroiditis. The presence of TPOAb and TgAb in our case can be explained by the fact that both Graves' disease and Hashimoto's thyroiditis are a continuum of an autoimmune thyroid disease [7].

The presence of hyperthyroidism and elevated TSI in our patient leads us to assume that the patient had Graves' disease. The evidence for Graves’disease was compelling, but the best confirmatory test for Graves' disease, RAIU could not be performed because of recent iodinated CT contrast use. Of note, most intravenous CT contrast preparations have a large amount of iodine load that saturates the thyroid gland for many weeks and interferes with traditional thyroid scanning and uptake testing. The radioactive iodine uptake is almost invariably elevated in Graves’ disease (>35%), whereas it is low in the active phase of subacute thyroiditis (<5%), clearly differentiating the two entities. An elevated thyroid-stimulating immunoglobulin titer supports the diagnosis of Graves' disease, but by itself lacks sufficient sensitivity or specificity [8].

Despite this limitation, the absence of any alternative diagnosis and resolution of symptoms with antithyroid medication further supports our diagnosis. Transient hyperthyroidism, as well as subsequent Graves' disease following subacute thyroiditis, is well-recognized clinical entity [9-13]. The simultaneous occurrence of Graves' disease with subacute thyroiditis was studied by Nakano Y et al. and they found seven patients (one male and six female) with subacute thyroiditis followed by Graves' disease during a period of 24 years between 1985 and 2008. The intervals between the onsets of subacute thyroiditis and Graves' disease were one to eight months (mean 4.7 months) [14]. Graves' disease presenting as painful and tender thyroid gland in the absence of thyroiditis is very rare. In the review of the literature, we found only three cases of Graves' disease presenting as a painful and tender thyroid gland in the absence of subacute thyroiditis.

Alves C et al. presented a case of a 10-year-old girl with painful and tender goiter. Hyperthyroidism with diffusely increased ^131 ^I uptake and presence TSI suggested a diagnosis of Graves' disease. She responded well to treatment with antithyroid medication [1]. Stanley JM et al. reported a case of 13-year-old girl presented with acute painful swelling of the thyroid gland and overlying erythema. She had an elevated RAIU, TSI, TPO Ab, and a normal sedimentation rate and leukocyte count. The tenderness of the thyroid gland as well as the symptoms and biochemical changes of hyperthyroidism resolved with propylthiouracil [2]. Jeong et al. presented a case of 25-year-old male who had complained of palpitations, muscle weakness, and a painful and tender goiter. His thyroid function test showed thyrotoxicosis. A 99mTc-pertechnate thyroid scan showed diffusely increased uptake. He responded well to treatment with methimazole and prednisolone [3]. One possible explanation of the extreme thyroid pain is unusual rapid gland growth with some restrictive element contributed by the features of autoimmune thyroiditis [1].

## Conclusions

A painful and tender thyroid gland is commonly associated with acute thyroiditis; its association with Graves' disease is unusual. A painful thyroid gland should not lead us to ignore the possibility of Graves' disease.
